# Ultrasonographic examination of the spinal cord and collection of cerebrospinal fluid from the atlanto-occipital space in cattle

**DOI:** 10.1186/s12917-015-0545-z

**Published:** 2015-09-02

**Authors:** Ueli Braun, Jeannette Attiger, Carina Brammertz

**Affiliations:** Department of Farm Animals, Vetsuisse-Faculty, University of Zurich, Winterthurerstrasse 260, CH-8057 Zurich, Switzerland

**Keywords:** Cattle, Ultrasonography, Spinal cord, Subarachnoid space, Cerebrospinal fluid

## Abstract

**Background:**

This study describes the ultrasonographic appearance of the atlanto-occipital space and ultrasound-guided collection of cerebrospinal fluid (CSF) in cattle. The atlanto-occipital space of 73 euthanized cattle (group A) and 14 live cattle with neurological disorders (group B) was examined in the sagittal and transverse planes using a 5.0-MHz convex transducer. Optimal ultrasonograms were frozen on the screen and various variables were measured in both planes using the electronic cursors. Puncture of the subarachnoid space was achieved using a spinal needle introduced in the median plane in a caudoventral direction while the spinal cord was viewed in longitudinal section. The examination of cerebrospinal fluid (CSF) was limited to gross evaluation and a red blood cell count.

**Results:**

The spinal cord and the subarachnoid space were seen in the sagittal plane in all cattle. In group A, the mean distance between the skin and subarachnoid space was 38.6 mm, the mean depth of the subarachnoid space dorsal and ventral to the spinal cord was 8.9 mm and 8.4 mm, respectively, the mean diameter of the spinal cord was 9.9 mm and the mean diameter of the dural sac was 26.9 mm. These measurements were similar on transverse images. For collection of CSF from the subarachnoid space, the spinal cord was viewed in longitudinal section. All CSF samples from group A were colourless and clear and the median erythrocyte count was 2.5/μl. Ultrasonographic findings and results of CSF analysis were similar in group B.

**Conclusions:**

Ultrasonography is useful for the visualisation of the spinal cord and facilitates the safe collection of CSF from the atlanto-occipital space in cattle.

## Background

The examination of cerebrospinal fluid (CSF) plays a major role in the diagnosis of central nervous system diseases in cattle. There are two sites from which CSF can be collected in cattle: the first is the atlanto-occipital (AO) space and the second is the lumbosacral foramen (LSF) [1, 2]. The exact site of needle insertion at both locations is determined by skeletal landmarks but puncture is carried out blindly without visualisation of the subarachnoid space [1–4]. A thorough knowledge of the anatomy of the atlanto-occipital space is a prerequisite for the successful collection of CFS. The atlanto-occipital space is bordered by the occiput cranially and by the atlas caudally and is covered by the atlanto-occipital membrane, various muscles, the nuchal ligament and skin dorsally [2]. The vertebral canal contains the spinal cord surrounded by three meninges. The outermost meninx is the dura mater, which is separated from the vertebral periosteum by the epidural space [5]. The middle meninx is the dura arachnoidea, which consists of three layers; the middle layer is referred to as the subarachnoid space [6]. The pia mater is the innermost meninx. The spinal cord is a cylindrical structure with a central canal, which is continuous with the ventricular system of the brain [6].

For collection from the atlanto-occipital space, the head is ventroflexed at a 90° angle and the needle is inserted at the intersection between the dorsal midline and the line connecting the cranial edges of the wings of the atlas [2, 4] or slightly cranial to the intersection [1]. The spinal needle is introduced into the subarachnoid space parallel to the longitudinal axis of the flexed head [1, 4]. The depth at which this space is reached is not exactly predictable, and the needle is advanced slowly and carefully and monitored for free flow of CSF by removing the stylet at regular intervals [2]. Puncture of the spinal cord must be avoided because it can lead to nerve damage or even death of the patient [2, 7]. Blind aspiration of CSF from the atlanto-occipital space frequently results in contamination of the sample with blood [8, 9], which can impair the diagnosis [10–14]. This emphasises the importance of the collection of uncontaminated CSF samples, which has been achieved in horses using ultrasonography. Two ultrasonographic techniques have been described in the horse [15] and both use general anaesthesia. In the first technique, the subarachnoid space is accessed from the dorsal midline while observing the median sagittal plane ultrasonographically. In the second technique, puncture of the subarachnoid space is achieved through a parasagittal oblique plane while monitoring a parasagittal oblique image. These techniques have also been used in the standing sedated horse [16]. Another study described collection of CSF between the first two cervical vertebrae using a parasagittal approach while observing the spinal cord on a cross-sectional image in the standing horse [17]. The results of those three studies showed that ultrasonography facilitated the precise puncture of the subarachnoid space and reduced complications and blood contamination of the sample. The purpose of the present study was therefore to describe the spinal cord and its surrounding structures ultrasonographically and to examine the feasibility of ultrasound-guided CSF collection in cattle.

## Methods

### Group A (euthanased cattle)

Group A included 73 cattle of different breeds (33 Brown Swiss, 22 Holstein Friesian, 13 Simmental, 5 other). They ranged in age from 1.2 to 13.5 years (median 4.3 years) and were euthanased for various reasons with an overdose of pentobarbital immediately before CSF was collected.

### Group B (live cattle with central nervous system disease)

Group B included 14 live cattle of different breeds (8 Brown Swiss, 3 Holstein Friesian, 3 other). They ranged in age from 0.2 to 10.3 years (median 2.5 years) and underwent CSF collection because of central nervous disease.

### Ultrasonographic examination and CSF collection in group A

**Fig. 1:**
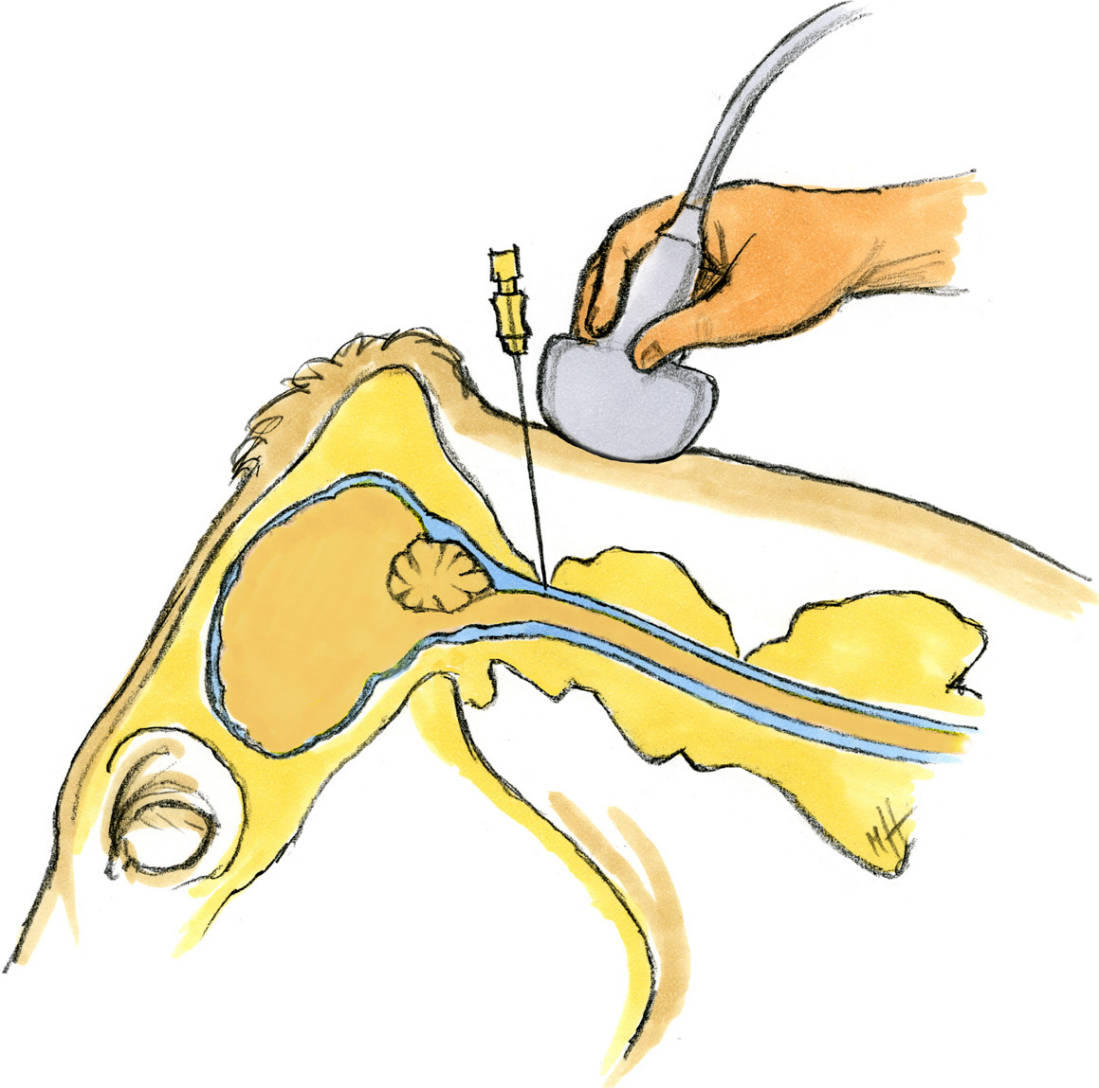
Schematic diagram of puncture of the subarachnoid space. Schematic diagram of puncture of the subarachnoid space for collection of cerebrospinal fluid. The diagram is based on MRI images of the head of a 10-year-old Simmental cow

The dead cattle were placed in lateral recumbency and the head was fixed in a normal position (head not ventroflexed, 0°) or in a mildly (about 30°) or moderately ventroflexed (about 45°) position. An attempt was first made to examine the spinal cord in a normal non-ventroflexed head position. When the resulting ultrasonogram was inadequate or when only a very short section of the spinal cord could be visualised, a mild to moderate degree of ventroflexion was applied to the head. A 15 cm × 10 cm area over the AO space was clipped and cleaned with ethanol and the area was examined ultrasonographically using a 5-MHz convex transducer (Logiq 7, GE Medical Systems, Glattbrugg) after the application of conductive gel (Aquasonic® 100, Parker Laboratories Inc.). The spinal cord and its surrounding structures were imaged and assessed in longitudinal and cross section. Optimal ultrasonograms were frozen on the screen and the following variables were measured in both planes using the electronic cursors: distance between the skin and the arachnoidea, depth of the subarachnoid space dorsal and ventral to the spinal cord, diameter of the spinal cord and diameter of the dural sac (made up by the depth of the subarachnoid space dorsal and ventral to the spinal cord and the diameter of the spinal cord). The length of the visible spinal cord was recorded. The measurements were repeated twice using newly frozen images in both planes, and means were calculated and used for analysis. The degree of ventroflexion of the head was recorded in all images. Puncture of the arachnoidea was achieved using a spinal needle (0.90 × 90 mm, Terumo®Spinal needle, Cosanum AG, Schlieren) introduced in the median plane in a caudoventral direction while the spinal cord was viewed in longitudinal section (Fig. 1). After penetration of the musculature and ultrasonographic visualisation of the tip of the needle in the subarachnoid space, the stylet was removed and 5 ml CSF was aspirated into a syringe and then transferred into an EDTA tube. If the collection was unsuccessful, the stylet was replaced and the needle withdrawn slightly or completely and re-introduced at a slightly different angle. When blood was aspirated, a new needle was used for subsequent attempts. The number of aspiration attempts was recorded.

### Ultrasonographic examination and CSF collection in group B

**Fig. 2:**
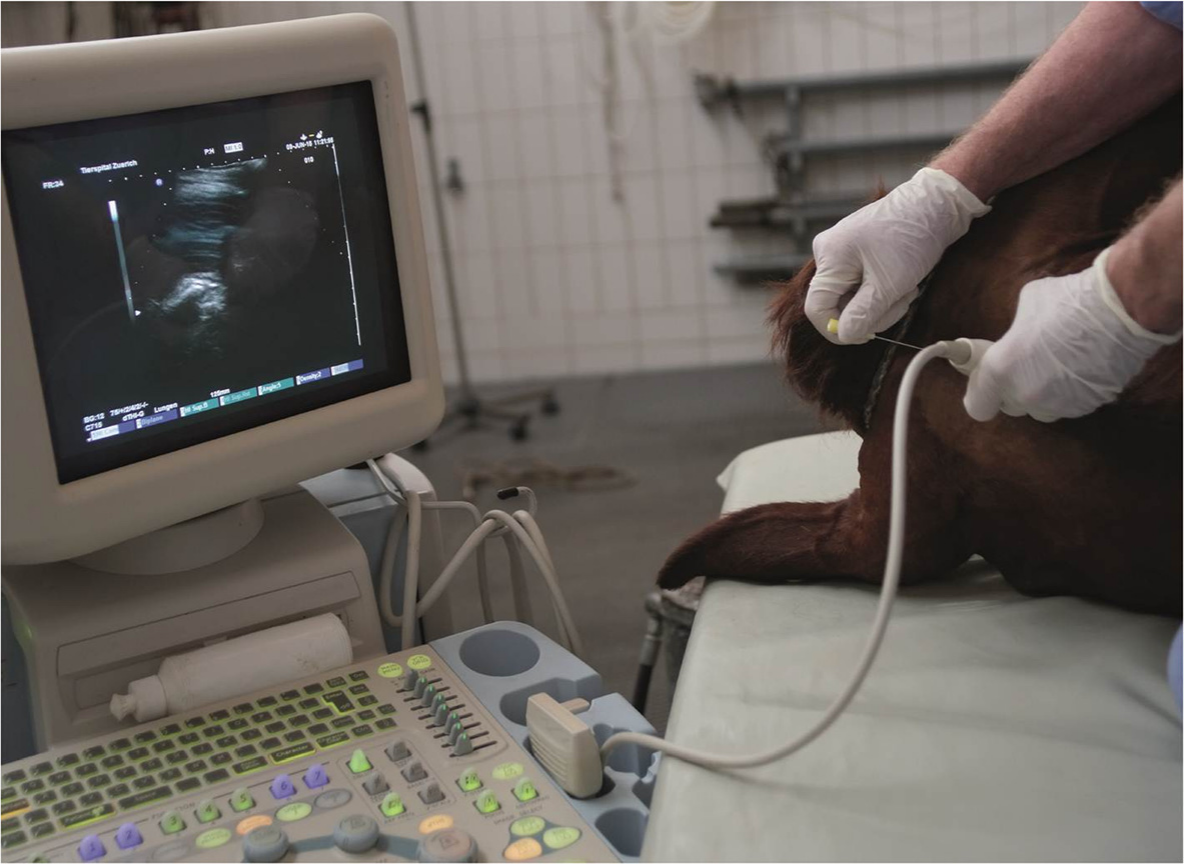
Collection of cerebrospinal fluid. Collection of cerebrospinal fluid in a sedated cow in lateral recumbency. The head and legs are fixed to the operating table. The fluid is collected from the atlanto-occipital space using a spinal needle and ultrasonographic guidance provided by a 5-MHz convex transducer

The cattle of group B were sedated using 0.07 to 0.10 mg/kg xylazine (Xylazin Streuli®, Streuli Pharma, Uznach) intravenously, followed by 0.05 mg/kg intramuscularly depending on the level of sedation. The sedated cattle were placed in lateral recumbency on a tilt table and all 4 feet and the head were secured (Fig. 2). The head was fixed to the table with a halter in mild ventroflexion (30°). The skin over the atlanto-occipital space was prepared and the spinal cord and surrounding structures examined ultrasonographically as described in group A. The area was then cleaned (Betadine® liquid soap, Mundipharma Medical Company, Basel) and disinfected (Betadine® standardised solution, Mundipharma Medical Company) and 5 ml lidocaine (Lidocain 2 % Streuli®, Streuli Pharma) was injected at the site of needle insertion for local anaesthesia. The spinal needle was inserted in the median plane as described in group A. The cows were returned to a standing position immediately after CSF collection, that is, within about 5 min.

### Examination of cerebrospinal fluid

In group A, the examination of CSF was limited to gross evaluation (colour, viscosity) and determination of the red blood cell count using a Neubauer haemocytometer. In group B, samples were grossly evaluated and the protein concentration and white blood cell count including differentiation were determined. These latter results were not relevant for this study and are therefore not shown.

### Statistical analysis

The statistical program STATA 12 (StataCorp LP, College Station, Texas, USA) was used to calculate means and standard deviations for data with normal distribution and to calculate medians for data with non-normal distribution.

### Approval of the study by an ethical committee

The study was approved by an ethical committee of the canton of Zurich, Switzerland.

## Results

### Ultrasonographic examination and collection of CSF in group A

**Fig. 3:**
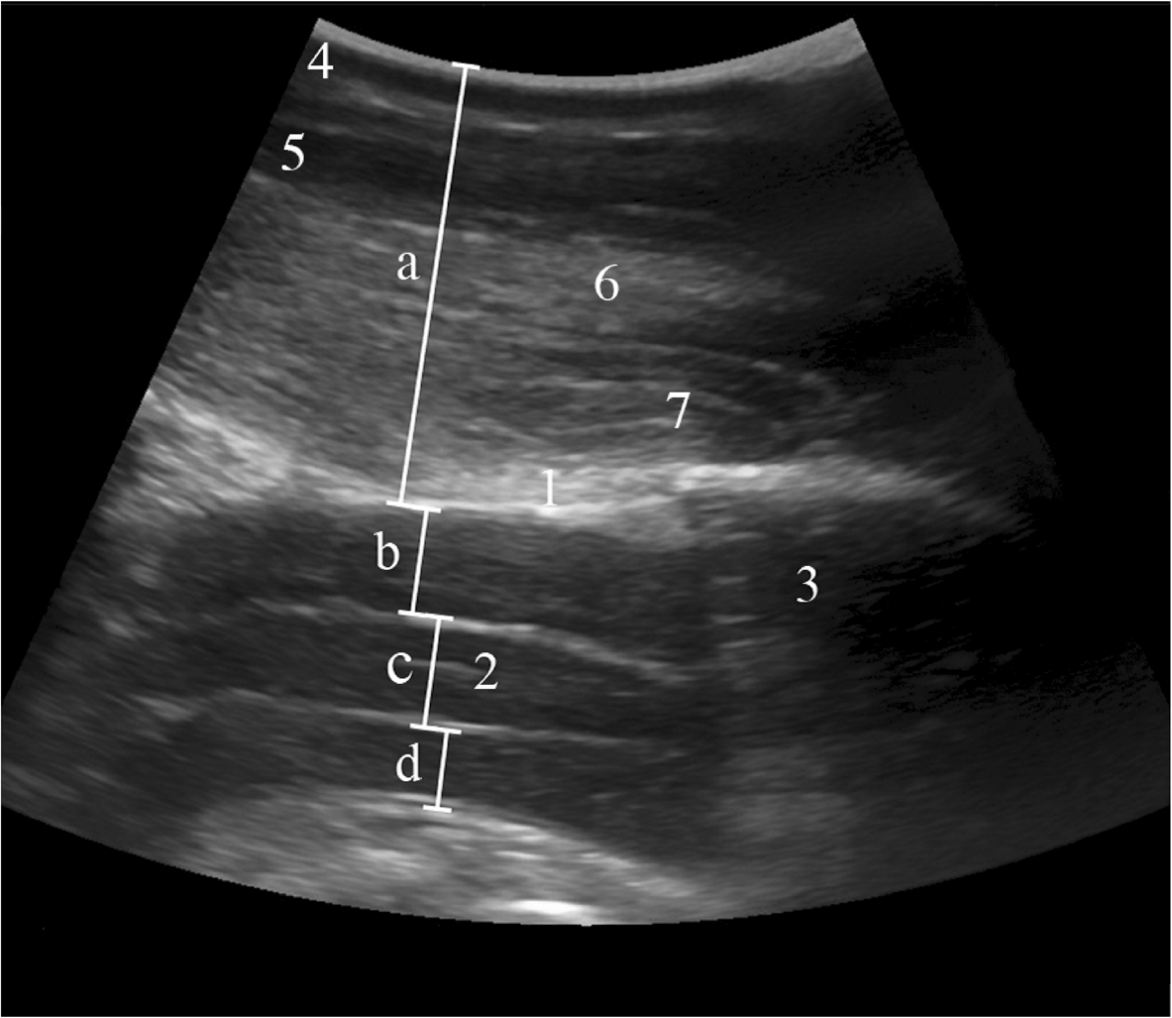
Longitudinal ultrasonogram of the vertebral canal. Longitudinal ultrasonogram of the vertebral canal in *a* 4.6-year-old Swiss Braunvieh cow at the level of the atlanto-occipital space immediately after euthanasia. Left is cranial and right is caudal. *a* Distance between skin and arachnoidea, *b* Dorsal compartment of the subarachnoid space, *c* Spinal cord, *d* Ventral compartment of the subarachnoid space, *1* Atlanto-occipital membrane, *2* Central canal, *3* Atlas, *4* Skin, *5* Nuchal ligament, *6* Major rectus capitis muscle, *7* Minor rectus capitis muscle

**Fig. 4:**
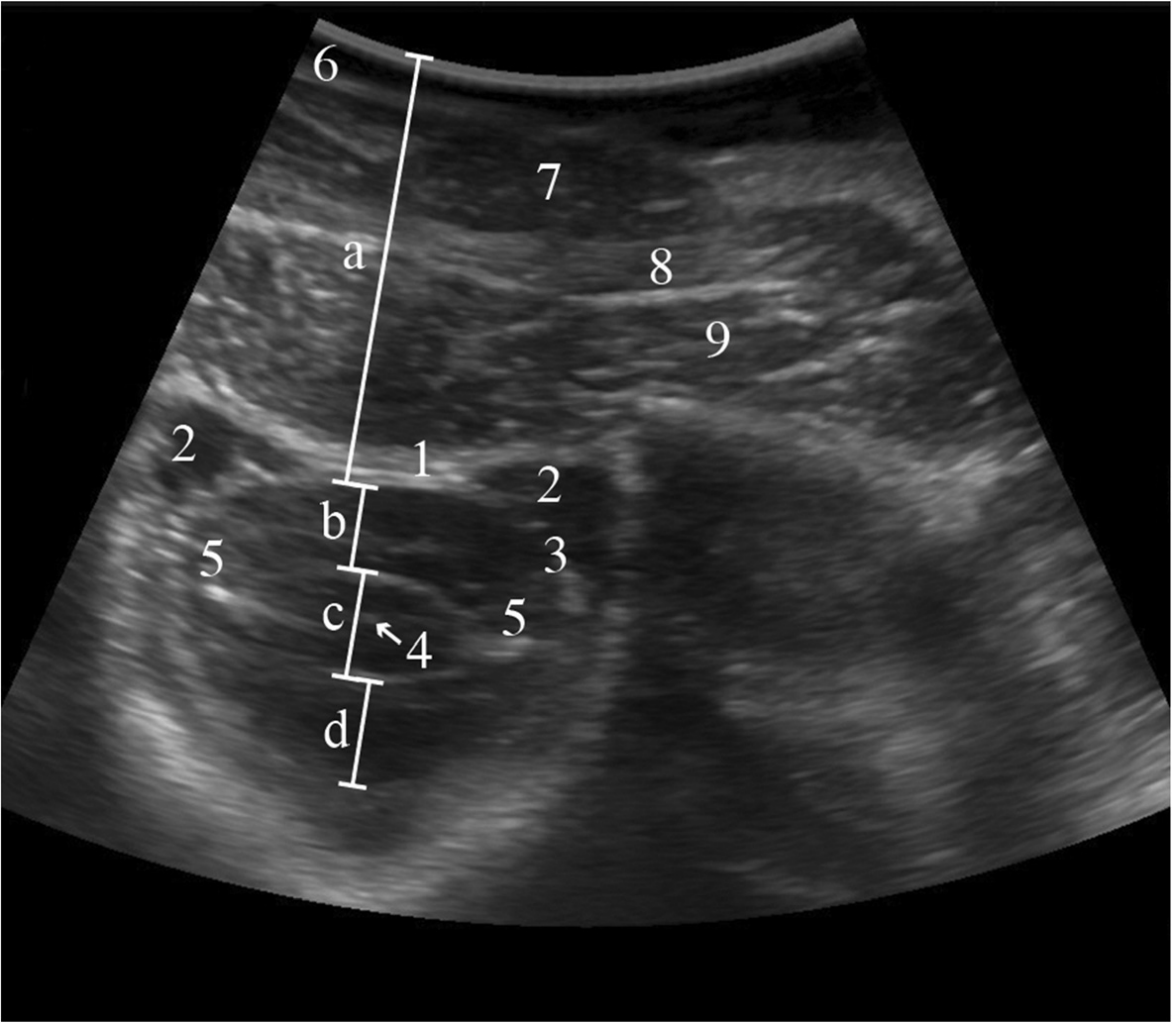
Transverse ultrasonogram of the vertebral canal. Transverse ultrasonogram of the vertebral canal in *a* 5.7-year-old Swiss Braunvieh cow at the level of the atlanto-occipital space immediately after euthanasia. *a* Distance between skin and arachnoidea, *b* Dorsal compartment of the subarachnoid space, *c* Spinal cord, *d* Ventral compartment of the subarachnoid space, *1* Atlanto-occipital membrane, *2* Venous sinus within the epidural space, *3* Dura mater and arachnoidea, *4* Central canal, *5* Denticulate ligament, *6* Skin, *7* Nuchal ligament, *8* Major rectus capitis muscle, *9* Minor rectus capitis muscle

The spinal cord and the surrounding structures could be seen in the sagittal and transverse planes in all cattle of group A. The structures identified in the area of the atlanto-occipital space from dorsal to ventral were the skin, nuchal ligament, parts of the rectus capitis dorsalis minor and major muscles, the atlanto-occipital membrane and the vertebral canal, surrounded by the hyperechoic dura mater. In the sagittal plane, the muscles appeared as echoic structures with longitudinal striations, and the nuchal ligament was hypoechoic. The spinal cord appeared as a hypoechoic band, and some areas had a heterogeneous internal structure (Fig. 3). The subarachnoid space surrounding the spinal cord was seen dorsal and ventral to the spinal cord and was anechoic to hypoechoic and sometimes had a heterogeneous internal structure. Blood vessels often seen dorsolateral and adjacent to the dura mater were interpreted as venous sinuses based on findings in the horse [15]. The spinal cord was circular and completely surrounded by the subarachnoid space in cross-section (Fig. 4). The hyperechoic denticulate ligaments between the pia mater and dura mater were often seen on both sides of the spinal cord. The central canal was frequently seen as a hyperechoic structure in the middle of the spinal cord. The pia mater appeared as an echoic line adjacent to the spinal cord. The dura mater and arachnoid membrane were also seen as a hyperechoic line but could not be differentiated.

Ventroflexion of the head was not required in 19 of 73 cattle (26 %) for ultrasonographic visualisation of the spinal cord and CSF collection. Mild ventroflexion of the head (≈30°) was used in 52 cattle (71 %) and moderate ventroflexion (≈45°) in two cases (3 %).

The mean distance between the skin and arachnoidea was 38.6 mm in the sagittal plane (Table 1). The mean height of the subarachnoid space dorsal and ventral to the spinal cord was 8.9 mm and 8.4 mm, respectively, the mean height of the spinal cord was 9.9 mm and the mean height of the entire dural sac was 26.9 mm. The spinal cord was seen in the sagittal plane over a distance of 43.1 mm. The measurements made in the transverse plane were very similar (Table 1).

**Table 1:** Ultrasonographic measurements of the vertebral canal at the atlanto-occipital space in euthanased cattle of group A (mm, mean ± standard deviation, median, range)

Variable	Section	
	Longitudinal	Transverse
Distance between skin and arachnoidea	38.6 ± 4	39.5 ± 4.2
	(30 – 52)	(32 – 52)
	*n* = 68^a^	*n* = 73
Depth of the subarachnoid space dorsal to the spinal cord	8.9 ± 1.6	9.2 ± 1.6
	(5 – 12)	(6 – 13)
	*n* = 67^b^	*n* = 73
Diameter of spinal cord	9.9 ± 1.2	10.1
	(6 – 13)	(8 – 15)
	*n* = 67^b^	*n* = 72^b^
Depth of the subarachnoid space ventral to the spinal cord	8.4	8.8 ± 1.8
	(4 – 11)	(5 – 14)
	*n* = 68	*n* = 73
Diameter of entire dural sac	26.9 ± 3	28.2 ± 3.5
	(20 – 34)	(21 – 40)
	*n* = 68	*n* = 73
Length of visible spinal cord	43.1 ± 10.3	
	(19 – 72)	-
	*n* = 67^b^	

^a^Could not be analysed in five cattle because of erroneous measurements and imprecise recording

^b^Could not be analysed in one animal each

The number of punctures required for CSF collection ranged from 1 to 5 (median, 1); one puncture was required in 48 cases (66 %), two in 15 cases (21 %), three in eight cases (11 %), and of the two remaining cases (2 %), one required four punctures and the other five punctures. In eight cases that required more than one attempt, haemorrhagic fluid was first aspirated, and in the remaining 17 such cases, no fluid could be collected at first, most likely because the needle was not in the subarachnoid space.

All 73 CSF samples were grossly colourless and clear. In the 72 samples that could be analysed, the erythrocyte count varied from 0 to 820/μl (median = 2.5 erythrocytes/μl). Twenty-two samples were free of erythrocytes, 36 samples had 1 to 10, 11 samples had 11 to 86 and 3 samples had 286 to 820 erythrocytes/μl. The 8 samples that were preceded by grossly haemorrhagic samples contained no or very few erythrocytes.

### Ultrasonographic examination and collection of CSF in group B

The ultrasonographic findings in the sagittal and transverse planes in the 14 cattle with central nervous system disorders were the same as in group A. Puncture of the subarachnoid space and CSF collection were achieved without difficulty in all cattle with the head in mild (30°) ventroflexion. Two attempts at CSF collection were required in two cows. Adverse reactions during needle insertion did not occur. Thirteen samples were colourless and clear and one was slightly turbid and had mild xanthochromia.

## Discussion

The spinal cord and surrounding structures could be imaged ultrasonographically in all cattle in the sagittal and transverse planes. In longitudinal section, the spinal cord appeared as a hypoechoic band characterised by the hyperechoic central canal and the hyperechoic pia mater visible on the dorsal and ventral surfaces. It was circular in cross-section, and the internal structure was partially heterogeneous in both planes as described in the horse [15, 16, 18]. The subarachnoid space surrounding the spinal cord was anechoic to hypoechoic, had a heterogeneous internal structure and was bordered by the hyperechoic pia mater and the hyperechoic arachnoidea and dura mater, but the latter two structures could not be differentiated. The ultrasonographic description of the subarachnoid space of the horse varies; the space was anechoic in one study [15] and had mildly echoic striations visible in cross-section but not in longitudinal section in another study [17]. The striations were interpreted as arachnoid trabeculae between the arachnoidea and pia mater. The internal structure of the subarachnoid space appeared heterogeneous in both planes in the cows of the present study. The mean height of the subarachnoid space dorsal and ventral to the spinal cord was 8.9 mm and 8.4 mm, compared with about 1.5 cm for the dorsal section in the standing horses [16]. This difference may relate to the larger body size of horses compared with cattle. Another possible explanation is that the horses were under general anesthesia and their heads were flexed, which might have increased intracranial pressure. In neonatal foals, the height of the dorsal section of the subarachnoid space ranged from 6 to 9 mm [19], and the difference between the two age groups suggested an association between height of the subarachnoid space and body weight.

The so-called freehand technique [20] was used for centesis in both groups. Positioning the needle so that it was aligned with the sagittal orientation of the sound waves posed a few problems initially, but it did not take long to master this technique and avoid puncturing blood vessels. The blood vessels dorsolateral to the spinal cord, which were interpreted as venous sinuses based on a study in the horse [15], were occasionally and inadvertently punctured at first, and the error was quickly recognised by blood emerging from the hub of the needle. A complication was reported in horses when the angle between the needle and dura was small, which resulted in the needle pushing the dura mater ventrally, instead of perforating it [15]. This complication is referred to as tenting phenomenon and has been described in human beings [21] and in another study on horses [16]; however, these authors did not feel that the angle of the needle relative to the dura mater was the cause. The tenting phenomenon increases the risk of accidental puncture of the spinal cord, but was avoided in horses by using ultrasonographic guidance [15]. The tenting phenomenon was encountered several times in the present study, but puncture of the spinal cord could be avoided.

Ultrasound-guided collection of CSF was successful in the first or second attempt in most cases. All but one sample were clear and colourless and all had no or minimal microscopic evidence of blood contamination. Compared with samples from horses, the erythrocyte count of CSF samples was higher [15, 16] or similar [17] in the cattle of the present study, but smaller than in samples collected using blind puncture without ultrasonographic guidance [8, 9]. A minimum erythrocyte count of about 2000 to 3000 cells/μl is required to render a CSF sample grossly discoloured or turbid [10, 11, 22], and blood contamination often goes macroscopically unnoticed [9, 11, 12].

In the live cows of group B, ultrasonographic guidance of the puncture was superior to blind puncture because only mild ventroflexion of the head and therefore only mild sedation was required. In contrast, blind puncture requires marked ventroflexion of the head of up to 90° [1, 2, 4], which results in stress, resistance to handling and possible respiratory depression in the animal. Furthermore, twitching movements, which are common during blind puncture of the dura mater, did not occur with ultrasonographic guidance. These movements are most likely caused by accidental puncture of the spinal cord.

## Conclusion

The spinal cord and its surrounding structures were readily identified using ultrasonography. Collection of CSF was successful in all cases and blood contamination of the fluid was avoided or kept to a minimum under ultrasound guidance. All collected samples could be used for diagnostic purposes. Furthermore, ultrasound guidance eliminated the need for marked ventroflexion of the head, which in turn minimised defensive reactions that commonly occur when the blind technique is used. Ultrasound-guided puncture of the subarachnoid space from the atlanto-occipital site is convenient and safe and therefore the method of choice for collection of CSF samples in cattle.
